# Sufficiency and healthcare emissions

**DOI:** 10.1111/bioe.13400

**Published:** 2025-02-04

**Authors:** Joshua Parker

**Affiliations:** ^1^ Lancaster Medical School Lancaster University, Health Innovation One Bailrigg Lancaster UK

## Abstract

In this paper, I am concerned with how healthcare systems ought to transition away from the greenhouse gas emissions that they have historically relied on to provide care. I address two questions in relation to this issue. The first is what emissions target should healthcare systems adopt? Second, is how should the burdens of mitigation be shared fairly in light of that target? I argue that sufficientarianism offers an attractive way to answer both of these questions because it is better situated to strike the right balance between healthcare benefits and the costs of mitigation than rivals. Sufficiency describes the view that what is important from the perspective of distributive justice is that individuals have enough. I argue that this ideal can be used to set a threshold of enough health from which an emissions threshold can be set. Once an emissions threshold is in place, this can be used to demarcate permissible from impermissible emissions in healthcare. In turn, the emissions threshold provides guidance on which emissions are liable to mitigation and when it would be fair for healthcare to shoulder the associated burdens. Permissible emissions, on the other hand, are necessary to secure sufficiency and so healthcare's mitigation responsibilities should be altered in light of this. I also discuss various alternative methods of setting an emissions target like net zero, zero emissions, emissions grandfathering and emissions egalitarianism. I point to several issues with these approaches.

## 1 INTRODUCTION

Historically, change in medicine has been characterised by addition. Medical power increases through scientific advances such that healthcare can utilise new technologies and increased knowledge to offer new treatments, new diagnostics, new diagnoses and so forth. Theories of distributive justice in healthcare concern themselves with how to distribute the benefits of these developments in medical knowledge and power fairly. That is, justice in healthcare has been interested in justified claims to the benefits of healthcare, and ensuring patients receive a fair share of the benefits of limited medical resources.

A new challenge for theories of justice in healthcare is present. This challenge arises from healthcare systems reliance on greenhouse gas (GHG) emissions: how can theories of justice in healthcare accommodate not addition but a transition to environmentally sustainable systems, and the burdens, rather than benefits, that entails? Instead of thinking about the *benefits* brought about by distributing medical resources, theories of justice in healthcare are having to consider how to distribute *burdens* as healthcare systems transition to low‐carbon, sustainable, models of care.

As a source of a significant volume of GHGs, healthcare is recognised as contributing to the threats of climate change. I take an institutional perspective on the locus of responsibility when it comes to reducing healthcare emissions. I use ‘healthcare’ and ‘healthcare system’ interchangeably to refer to the organised efforts of societies to promote health, prevent disease and provide medical care. Modern healthcare systems have a complex organisational pattern and are formed of various organisations like hospitals and clinics, as well as individuals like managers and healthcare staff. It is this collective that I am concerned with when thinking about healthcare's responsibilities to address climate change.

Globally, healthcare systems are thought to be responsible for around 4% of GHG emissions^1^, though each healthcare system's contribution is different. Consequently, healthcare is tasked with reducing GHG emissions. One problem, however, is the burdens associated with addressing climate change and shifting away from the GHG emissions that healthcare systems have historically relied upon to meet its goals like protecting and promoting health. Three main burdens are associated with tackling climate change: mitigation, adaptation and compensation.^2^ Whilst each is potentially relevant to healthcare systems, as efforts to reduce GHG emissions form the bulk of how healthcare systems have responded to the threat of climate change so far, I focus on mitigation here.

This paper is concerned with two questions of distributive justice regarding healthcare emissions: (1) what goal should healthcare systems adopt when reducing GHG emissions? And (2) what is a fair share of the burdens (and benefits) of healthcare systems adopting policies that meet this goal? The climate target question is important because it determines the extent of emissions reductions for a healthcare system. Take a target of net zero where some GHG emissions are permissible as long as they are balanced by enhancing carbon sinks. Net zero demands far less of healthcare than a target of zero emissions whatsoever. I discuss these positions in more detail later. The second question is concerned with how the target is achieved fairly.

These questions raise two methodological issues. First, since climate change mitigation entails burdens and theories of justice in healthcare are primarily concerned with benefits, a key methodological question is how to integrate a theory of justice in healthcare with climatic responsibilities. In particular, how can healthcare simultaneously meet its goals like promoting health and treating disease whilst also doing its fair share to address climate change? An alternative methodology would be to adopt separate, climate‐specific principles to determine healthcare's mitigation responsibilities; for example, a polluter pays principle. A second, related methodological issue arises regarding the question of the appropriate target. Should the climatic goals that healthcare aims for, and how they are achieved, be examined independently or do they share a common normative grounding? Should these two questions be addressed simultaneously or separately?

An example helps to highlight these two methodological issues. Take a polluter pays principle. This states that polluters ought to mitigate because of, and to the extent to which, polluters contribute to a problem. Polluter pays offers a common normative grounding to both the target question and the fair shares question. A polluter's emissions should be eliminated on a polluter pays principle, and doing so means the polluter has done their fair share. The target is based on a polluter's emissions, as is their fair share, and so both questions are addressed by the same principle. Nevertheless, allocating mitigation responsibilities on the basis of being a polluter leaves this principle insensitive to the wider goals of justice, in particular, the goals of healthcare because healthcare's goals are irrelevant to the question of whether healthcare is categorised as a polluter. Hence, polluter pays offers a separate principle to a theory of distributive justice in health and is a non‐integrated principle.

Here I take an integrated approach to both the just target and the fair shares questions. I do not defend an integrationist methodology, rather I hope that my arguments provide an example of how this might work. I argue that sufficientarianism is particularly well suited to strike a balance between the distribution of healthcare benefits as well as the burdens of mitigation compared to rivals.

The paper is formed of two sections one devoted to each of the two questions above. In the first section, I consider alternative ways to set a mitigation target for healthcare and point to specific problems with each method. I then lay out the sufficientarian perspective pointing to several attractive properties in the sufficiency view's ability to navigate problems left by rival views. Following this I move to consider what a fair share of the burdens of climate change mitigation are for healthcare. My position is that sufficientarianism leaves healthcare with a budget of permissible and exempt emissions. Any emissions beyond this budget are liable to mitigation. This framework for distinguishing permissible from impermissible emissions can determine a fair share of mitigation burdens for healthcare allowing it to reconcile its valuable goals with tackling climate change.

## 2 WHAT GHG EMISSIONS TARGET SHOULD HEALTHCARE AIM FOR?

In this section, I address the question of what mitigation target a healthcare system should adopt. First, however, I briefly cover the idea that mitigation in healthcare entails burdens. This is not a universally accepted claim.^3^

As mentioned above, mitigation refers to actions that limit the impact of GHGs on global surface temperatures. There are generally two ways agents can mitigate. One is to directly reduce GHG emissions, and the second is indirect through enhancing carbon sinks or offsetting emissions. I claim that mitigation is burdensome because it places responsibilities on some agents, and to discharge those responsibilities they will have to shoulder costs in taking action to address their GHG emissions. Now, some may object to this, claiming that mitigation in healthcare has longer‐term and globally distributed benefits in terms of protecting health and reducing healthcare costs. For example, as alternatives to fossil fuels are increasingly affordable and scalable, there are opportunities to reduce healthcare's reliance on fossil fuel‐generated energy.^4^ More renewable energy has downstream benefits by reducing healthcare's overall energy expenditure. Furthermore, as mitigation tackles climate change, and climate change threatens health, we can protect health by moving healthcare systems away from fossil fuels. Better to call mitigation an ‘investment’, so the argument goes, not a burden.

I do not deny that there are benefits to mitigation. Moreover, I recognise the political advantages to naming the costs of mitigation ‘investments’ rather than burdens to help accelerate the transition to an environmentally sustainable healthcare system. Reducing the GHG emissions of healthcare is, however, thought to go far beyond energy systems and to entail fundamentally altering how, where and crucially *what* healthcare is provided.^5^ Widespread change across healthcare systems is thought to be critical for reducing GHG emissions and this includes: changing how healthcare is structured and delivered, changes in the norms and culture of healthcare and behaviour change amongst professionals and patients in terms of how they access healthcare, what is offered and how medicine is practiced.^6^ So the main burdens arise from the structural changes, new technologies, foregone consumption opportunities, attitudinal and practice changes in healthcare, as well as investments, that are necessary to reduce emissions. The problem then is that investments take too narrow a view on the costs that a healthcare system undertakes to minimise its emissions.

Not all burdens are investments and not all costs are financial. Changing to low‐carbon inhalers offers one example.^7^ Switching a patient's inhaler to one which contributes less to climate change may be inconvenient requiring additional appointments and reviews, as well as potentially leading to the management of their lung condition, temporarily, worsening. Such switches are thought to have lower overall GHG emissions and there may be health benefits in the future through reducing the threats of climate change. But for the patient being asked to switch inhalers, it hardly seems right to call this an ‘investment’. Rather, they are being asked to accept, albeit small, burdens.

There are potentially benefits to addressing healthcare GHG emissions, but as healthcare systems act, we must be mindful that those actions may carry costs. So, there is a question of how those costs (or investments) and any benefits are distributed fairly. One important aspect of answering this question is addressing what emissions target a healthcare system should adopt. Let us first consider the question of the appropriate goal of climate policies in healthcare then. A key aspect is whether the target should be developed internally or externally to healthcare. Should there be a generic target formulated independently of healthcare's purpose which is later adopted by healthcare, or should the target come from healthcare itself? Two methods to determine the appropriate target result: one which is sensitive to the purpose of healthcare and one which sets the target independently of healthcare. I explore the latter method first.

### 2.1 Independent methods of setting healthcare's mitigation target

Three commonly discussed approaches to mitigation set a target independently of healthcare: zero emissions, net zero and emissions egalitarianism. I discuss each approach. The primary issue with methods that are insensitive to the goals of healthcare is that they tend to strike the wrong balance between sufficient emissions reduction and ensuring that healthcare can fulfil its valuable goals.

Zero emissions is considered briefly. This is because producing no emissions at all is an unrealistic target for healthcare, at least for now. It may well be that eventually zero emissions is feasible. But as some treatments rely on GHGs directly, like certain inhalers and volatile anaesthetic gases,^8^ even a complete shift away from fossil fuels would still leave residual emissions from these medicines.

Emissions egalitarianism garners support from many philosophers.^9^ In essence, this view claims that the relevant good to be distributed is emissions as opposed to welfare, capabilities or something else. Emissions are then distributed on an equal *per capita* basis. Egalitarians may have other methods of setting a target for GHG emissions reductions based on metrics other than emissions. But emissions egalitarianism is one common way that a global budget of emissions is determined.

To set the budget, the emissions egalitarian must first determine what global surface temperature rise they are willing to accept. The precise details involve complex empirical issues as well as normative considerations of what level of temperature rise is considered dangerous. To help illustrate consider the following forecasts from the Intergovernmental Panel on Climate Change (IPCC). The IPCC claim that, from 2020 onwards, to have a 50% chance of limiting global warming to 1.5°C leaves a remaining carbon budget of 500 Gigatonnes of CO_2_ equivalent. For a 67% probability of staying within 2°C leaves a budget of 1150 GtCO_2_, according to the IPCC.^10^ Emissions egalitarianism states that whatever emissions budget we have, based on whether we opt for 1.5°C, 2°C or something else, should be shared equally. As the absorptive capacity of the atmosphere is seen as a global commons, rights to emit GHGs are divided equally.^11^ This appears to be self‐evidently fair as no individual has a greater claim to the atmosphere than anyone else. The result is equal *per capita* emissions.^12^

It is not immediately obvious how an equal *per capita* share of emissions translates into an emissions target for healthcare systems. Broadly we could either allocate a budget to each individual, or states could be provided a GHG budget based on equal *per capita* emissions. But the issue is how to go from this allocation to one for healthcare.

A further consideration for resourcist egalitarian views like emissions egalitarianism is that individuals’ ability to convert resources into welfare differ.^13^ Individual's healthcare needs vary as do their capacities to benefit from healthcare interventions that cannot be provided without producing emissions. Allocating emissions on a *per capita* basis, potentially overlooks that an equal share of emissions may be insufficient to meet healthcare needs fairly. This could be avoidable, depending on how emissions are distributed once we have set a *per capita* share. For instance, a state could be allocated a share of emissions and then redistribute those emissions such that entitlements to healthcare were met fairly. Of course, if further redistribution of emissions beyond the initial *per capita* allocation is required to meet individual's just entitlements, then we may question the fairness of, and the basis for relying upon, emissions egalitarianism in the first place.

The problem for zero emissions and emissions egalitarianism then is that they are insufficiently sensitive to the valuable role that healthcare plays. Emissions egalitarianism risks that burdens are distributed unfairly or requires other distributive principles to further allocate emissions fairly. Net zero as an emissions target, on the other hand, faces the opposite problem. It has the potential to be too lenient towards healthcare when reducing emissions by not specifying a precise target but rather by describing a mitigation procedure.

Net zero represents a ledger where the goal is to ensure an agent's aggregate emissions are zero.^14^ GHG emissions reductions and by enhancing carbon sinks, or offsetting can all contribute to achieving net zero. Under net zero, agents have significant freedom to decide how to balance their books helping to explain why this has become such a popular organising framework. Indeed, many healthcare systems have committed to net zero.^15^ Instead of specifying a certain level of reduction or an emissions target then, net zero offers a balancing framework. It is only the date that net zero is to be achieved by that is concretely specified.

Net zero, as well as being politically palatable, has the advantage of allowing healthcare to continue to use GHG emissions to provide care, as long as these are eventually balanced by offsets. Unlike zero emissions and emissions egalitarianism, net zero can therefore be sensitive to the goals of healthcare. Healthcare can go ahead and emit GHGs to treat disease, ameliorate symptoms and such like, it just needs to make sure those emissions are sufficiently offset. But this balancing process is potentially opaque leaving the possibility that emissions balance only on paper. The flexibility afforded by net zero can facilitate ‘accounting failures’.^16^ That is, net zero provides a cover to give the impression emissions are neutralised when in reality they are not. In theory, net zero enables a process of balancing emissions whereby healthcare can do its fair share whilst simultaneously reducing the impact of its emissions, but the risk is that in practice healthcare ends up falling short of meaningful mitigation efforts.^17^

### 2.2 Accounting for the goals of healthcare in setting a mitigation target

I now discuss two methods of setting an emissions target for healthcare which account for the goals of healthcare. Rather than follow a nationally or internationally determined target like net zero, healthcare organisations could develop their own trajectory according to the specifics of healthcare. I dwell briefly on an account known as ‘grandfathering emissions’. I then advocate for an ideal of sufficiency.

A view which has had some political support is grandfathering.^18^ Emissions grandfathering is the idea that previous levels of emissions should serve as a guide to future emissions targets. Since healthcare has historically produced a large volume of emissions to provide goods like treating disease and care for the sick, on an emissions grandfathering view, healthcare should continue to have a substantial carbon budget. At the extreme, one might conclude that healthcare's emissions should remain unchanged because of healthcare's historic carbon footprint. But milder versions of grandfathering could still justify a large emissions budget for healthcare, even if only temporarily in a transition to lower emissions.

Like net zero, grandfathering has the potential to demand too little from healthcare as societies take action on climate change. At worst grandfathering gives healthcare a free pass on mitigation. At best it risks leaving a gap between what healthcare could do to reduce emissions and what is being asked of it. A key criticism of grandfathering is that it perpetuates the injustices of climate change, and this issue is reflected in healthcare.^19^ Grandfathering can perpetuate a pattern whereby those who contribute the least to climate change will continue to suffer the most. Further, by locking in historical emissions patterns, grandfathering prevents the redistribution of emissions to healthcare systems in developing countries preventing them providing care on a par with wealthy countries.^20^

An alternative view that is capable of accounting for the purpose of healthcare is sufficientarianism. Where sufficientarianism is particularly useful is in setting an emissions target for healthcare systems and ensuring healthcare does its fair share through the way it deploys thresholds. I further discuss the advantages that the sufficiency view holds over alternatives as well as showing how the two thresholds it leaves (the health threshold and the emissions threshold) can determine a fair share of mitigation burdens for healthcare in the next section. First, I lay out the basic idea behind sufficientarianism and explain how it corresponds to an emissions target.

Sufficientarianism describes a family of views that share the idea that what matters from the perspective of social justice is that everyone has enough.^21^ Two theses capture a sufficientarian pattern of distribution.^22^ The positive thesis is accepted by all sufficientarians, and it states that it is especially morally important for individuals to reach a threshold of enough. This is the key intuition captured by sufficientarianism, that it is unjust when individuals fall below a threshold of enough. The second thesis endorsed by sufficientarians is either the negative thesis or the shift thesis.^23^ The negative thesis claims that above a threshold of enough, inequalities are irrelevant to justice and no further distributive concerns apply. The shift thesis is weaker than the negative thesis and states that above a threshold of enough different distributive criteria apply as compared to below the threshold.

Through the sufficientarian's reliance on a threshold of enough, it can set an emissions target for healthcare indirectly. The sufficientarian is interested to know whether a specified volume of GHG emissions is enough to provide for basic needs, a decent life, some set of capabilities, or another metric of justice. So, to distribute emissions in a sufficientarian fashion we need to know ‘sufficient for what?’. We cannot simply stipulate some volume of emissions as enough without reference to a more fundamental goal of distributive justice which, as I discuss further later, refers to a threshold of enough. We cannot know, for example, whether 5 megatonnes of carbon dioxide equivalent or 25 megatones is enough without setting a threshold which we have weighty reasons to secure.^24^

Setting a threshold of emissions and hoping this is enough to meet the demands of justice fails to recognise the instrumental value of emissions. Rather, the sufficientarian threshold references a more fundamental demand of justice and from this we ask which emissions are necessary to achieve sufficiency.^25^ Starting with a sufficiency threshold we can work indirectly to an emissions target. In other words, some volume of emissions corresponds with sufficiency meaning we have two thresholds: a sufficiency threshold (which I call the health threshold later) and from this, an emissions threshold. The key question then is: ‘to secure sufficiency, how much carbon does healthcare *need*?’. Utilising increased scientific understanding of the emissions from healthcare we can calculate the emissions required to secure sufficiency. This emissions threshold forms the target for healthcare systems.

Two issues in relying on sufficientarianism to set an emissions target are worth discussing. One is how to set the sufficientarian threshold and second is the distributive principles used above and below the threshold.^26^ How each of these is answered has implications for what the precise target and emissions budget is. My comments on these will necessarily be brief for these details do not change my core claim which is that sufficientarianism, by setting a threshold of enough is able to set an emissions target and offers advantages over its rivals. The advantages are discussed in the next section.

Where to place the sufficientarian threshold is the subject of much debate and sufficientarians defend various thresholds.^27^ Similarly, philosophers debating climate change will defend thresholds inspired by sufficientarian considerations. For example, Simon Caney talks about ‘emissions required for [people] to attain a minimal decent standard of living’.^28^ Steve Vanderheiden refers to ‘emissions sufficient to allow for … basic human functioning’.^29^ There are two important considerations for a threshold of enough, however. The threshold ought to be unambiguous and non‐arbitrary.

Take the threshold suggested by Rodger Crisp of 80 years of good quality life.^30^ Crisp cannot be accused of being vague in where the threshold is placed, but we might feel that 80 years is somewhat arbitrary. On the other hand, a decent standard of living as suggested by Caney, or Vanderheiden's threshold of fulfil basic human functioning do not seem arbitrary but they are more ambiguous.

David Axelsen and Lasse Nielsen point out that for the threshold to be plausible, we must be able to justify the positive thesis, where bringing people up to the threshold is particularly important as well as the negative thesis where disadvantages above the threshold are less morally concerning.^31^ This is important because if the threshold is very low, for instance at survival, then the positive thesis is much easier to justify, but the negative thesis looks particularly harsh. Similarly, with a higher threshold, the negative thesis is easier to justify, but we may disagree with the positive thesis for every sub‐threshold benefit when the threshold is high. Axelsen and Nielsen argue that the threshold should therefore be determined by ‘thick’ normative concepts in the sense that we can articulate both a description of a morally concerning situation as well as evaluating it. Caney and Vanderheiden's thresholds are thick as a decent standard of living or basic human functioning are both descriptive but we also have moral reasons to care about such thresholds.^32^

The challenge of where the sufficiency threshold is set may be easier in healthcare, however, as it seems obvious that health is the relevant metric.^33^ Some level of health is important for one's ability to lead a decent life and at least some healthcare will be necessary to secure enough health. ‘Enough health’, or a health threshold of ‘sufficient health’, however, is not a complete answer. How we define and deploy concepts like health, and parallel concepts like disease, illness, malady and so forth, impact the threshold. In brief, a threshold based on health as biostatistical normal functioning will be quite different to one where health is associated with meeting one's goals or flourishing.^34^ This points to another issue regarding metric. Even if we accept a certain view of health, whether we see health as a basic need, as featuring within a decent life or as a capability will also shift the threshold.^35^

In spite of these issues, I assume that health is the relevant currency when it comes to a threshold of enough for a healthcare system. Health should be understood in accordance with the functional states of human bodies. This is so that a continuum of health states can more easily drawn up and a threshold on that continuum be clearly drawn. However, such functional states should be viewed broadly as those that we have reason to value, because they are necessary for a decent life. Whilst this specification of the threshold of sufficient health in terms of bodily function that is necessary for a decent life is somewhat vague in practice, it should not be a particularly controversial threshold and as thick conception of health ought to suffice for this paper. The emissions threshold then, is the minimum amount of emissions that is necessary for healthcare systems to secure sufficient health for a decent life.

What if it turns out that the emissions budget for healthcare surpasses the global emissions budget needed to stay within a certain temperature threshold? In so far as crossing this climatic threshold is seen as posing an unjustified threat to others, we are left with a challenging question. What needs to be decided is whether there ought to be further adjustments to the sufficiency threshold based on the global emissions threshold or not. This goes back to the issue of how the positive and negative thesis are justified by different thresholds. The basic idea is that setting a high sufficientarian threshold may result in an inflated emissions threshold. On the other hand, setting the threshold low will tighten the emissions budget; but a threshold that is too low and particularly austere makes the negative, or shift thesis, harder to justify as individuals have lives barely worth living. Broadly, one option is to bite the bullet and argue that if the necessary emissions for healthcare to meet the demands of justice exceed climatic thresholds, then this is a price that we must accept and shift the climatic threshold. Alternatively, the sufficientarian threshold could be altered. I discuss this further in the next section.

A final question concerns how we allocate above and below the threshold. This issue comprises several further questions. What principles should determine how medical resources are distributed above and below the threshold? Should we endorse the negative or the shift thesis mentioned above? Should individuals below the threshold be given lexical (absolute) priority, or rather weighted priority compared to those above? What is the value of securing sufficiency at the threshold? Like the question of setting the threshold, this is an important issue for fully fleshing out a sufficientarian distribution of emissions. I simply note these questions here, however. Clearly endorsing the negative thesis above the threshold, for example, will leave fewer emissions than accepting a distribution that includes those above the sufficiency threshold. Nevertheless, accepting that there may be different answers to these does not undermine the primary point which is that setting a threshold of enough can be used to guide an emissions target in healthcare.

## 3 A FAIR SHARE OF THE BENEFITS AND BURDENS OF MITIGATION FOR HEALTHCARE

The proceeding section defends two thresholds. One is the sufficientarian threshold of enough or the ‘health threshold’, the second is an emissions threshold that is instrumental to securing sufficiency. To begin this section, I discuss the merits of a sufficientarian approach in order to set up the argument for how the ideal of sufficiency can also answer what a fair share of the burdens of tackling climate change is for healthcare. In short, the emissions threshold demarcates permissible from impermissible emissions. The latter are liable to mitigation and signal where healthcare ought to shoulder mitigation burdens.

Let us first recap the problems that other approaches have to help make clear the challenge that a sufficientarian account of an emissions threshold must meet. So far, I have only suggested that sufficientarianism could be used to set an emissions threshold, hence it is important to examine why we ought to adopt this method. In setting an emissions target for healthcare there are three main challenges left by other accounts.

i. Avoiding the status quo. Grandfathering and net zero both result in potentially unambitious and unjust emissions targets.
ii. Sensitive to the goals of healthcare. Emissions egalitarianism and zero emissions are insensitive to the goals of healthcare. The risk is that healthcare is left with insufficient emissions to achieve its valuable role.
iii. A fair share of the burdens of decarbonisation. How the target is set has the potential to unfairly distribute burdens towards those with the greatest health needs who tend to be most disadvantaged.

Whilst keeping these problems with rival accounts in mind, a helpful place to start is with the question of under what conditions are GHG emissions justified and thereby permissible? After all, emissions contribute to climate change and in turn the threats it poses. Emissions do not discriminate in asserting their effects based on whether they were generated in the pursuit of basic goods or frivolous luxuries. Emissions then must be justified.

One defence of the idea that some GHG emissions are permissible is to argue that there is a limit to what individuals can be expected to sacrifice in minimising the threats posed by their emissions.^36^ That limit is those emissions which are released in the pursuit of subsistence. When Henry Shue distinguished between luxury and subsistence emissions, he was pointing out that we should not treat all emissions equally when it comes to mitigation, and that those emissions which are necessary to meet basic needs should be treated differently.^37^ In essence, Shue places a constraint mitigation through the concept of subsistence emissions. Subsistence emissions, unlike luxury emissions, are permissible and thereby exempt from mitigation.

Göran Duus‐Otterström cautions that the mere fact that emissions are devoted to subsistence is not enough to make them exempt, however.^38^ Wealthy individuals have subsistence needs and produce emissions to secure them, but that does not make them subsistence emissions. This is because of the substantial differences that some have in their ability to mitigate their emissions, including those that are to meet subsistence. For instance, the wealthy still need to use energy to cook food. But since the wealthy have vastly more opportunities to minimise these emissions, we should not take it for granted that these emissions are subsistence. So, it is important not just to consider the ends to which emissions are instrumental, but agents’ capacity to mitigate their emissions, including subsistence. To be permissible, and exempt from mitigation, Duus‐Otterström argues that agents must have exhausted all reasonable steps to ensure emissions are the minimum necessary to secure subsistence. Even if emissions do pose threats through climate change, claiming that they are the minimum that one could reasonably emit to secure a decent life offers a plausible defence.

As a constraint then, the concept of subsistence emissions is useful in placing a ceiling on permissible emissions. To be permissible then, on my view, GHG emissions must be the minimum necessary to secure a threshold of a decent life. Emissions that provide benefits below a threshold of enough and that agents have taken reasonable action to minimise are permissible and should be treated differently from those which are not necessary to secure enough. These are the necessary and jointly sufficient criteria for permissibility and exemption.

The health threshold is based on the sufficientarian idea that it is especially morally important to secure enough health. An emissions threshold corresponds to the idea of securing enough. But for those GHG emissions to be exempt from mitigation burdens, we must also ensure they are the minimum necessary and that there is nothing further we could reasonably expect healthcare to do to reduce those emissions. The emissions threshold then is not static and should shift in response to healthcare's available options for minimising emissions. If new technologies mean that healthcare can be provided with fewer emissions, in the absence of competing considerations, this ought to ratchet down healthcare's emissions threshold.^39^

Assessments reasonableness when healthcare is minimising emissions should reference the health threshold as well as other important goals like efficiency. For example, if a treatment offers a relatively minor reduction in emissions compared to an alternative, equally effective and vastly cheaper treatment, then the latter should be preferred. Excessive costs may undermine healthcare's ability to secure the health threshold through opportunity costs and so it would be unreasonable for healthcare to accept large mitigation costs, especially if the emissions reductions were minor. Similarly, imagine replacing ambulances used for patient transport with bicycles. Whilst this would minimise the emissions necessary to transport patients to hospital, it is highly ineffective and again may risk individuals securing the health threshold so would be unreasonable.^40^

There are several advantages to a sufficientarian method of setting the emissions threshold. For one, it is sensitive to the role of healthcare. In so far as healthcare performs tasks that are instrumental to securing enough health for a decent life, the sufficientarian threshold accounts for this. As sufficientarianism is primarily concerned with those below a threshold of enough, defending an emissions target that is instrumental in securing enough means that any burdens of mitigation are unlikely to fall on the least advantaged. As those who have the greatest health needs tend to be the least advantaged, and as the least advantaged tend to have contributed the least to climate change, by allocating GHG emissions based on achieving sufficiency, which tends to focus on the disadvantaged, we can avoid unfairness in the distribution of the burdens associated with climate change mitigation. Since GHG emissions are the minimum necessary to secure a decent life, this also avoids the problems of net zero and grandfathering where healthcare systems can continue to emit unabated. As I discuss momentarily, as strict limits are placed on emissions that are considered permissible, healthcare is left liable for various emissions over a threshold of those necessary to secure a moral minimum.

### 3.1 Permissible and impermissible emissions

I have argued that the emissions threshold demarcates a distinction between healthcare emissions which are permissible and exempt from mitigation and impermissible emissions which are liable. Permissible emissions are those below the emissions threshold, these are determined by assessing which emissions are the minimum necessary for healthcare to secure sufficiency. Any remaining healthcare emissions are therefore above the emissions threshold and ought to be subject to mitigation and any costs this entails. Excess emissions above the threshold include those used for medical benefits above a threshold of sufficiency. Furthermore, those emissions that, whilst contributing to securing sufficiency, can nonetheless reasonably be reduced are also above threshold.

It is worth emphasising that this demarcation between permissible and impermissible should not be taken to correlate with the positive and negative thesis of sufficientarianism, respectively. Of course, it is tempting to see impermissible emissions above the threshold as reflecting the negative thesis, because once sufficiency is reached there is no need for further emissions. Whilst this does capture the sufficientarian ethos, as I am rejecting resourcist sufficientarianism, this rests on a confusion. Sufficiency regards a threshold of a currency like a decent life. The health threshold, however, corresponds to a second separate threshold of necessary emissions, instrumental in securing the health threshold. Below the emissions threshold, there are several ways that emissions could be used to secure sufficiency. It may be that some do advocate the negative thesis and that above a threshold of sufficiency there is no need for further distribution in which case the sufficiency threshold and the emissions threshold are the same. However, we may, for example, use some emissions for individuals above the sufficiency threshold who are at risk of falling beneath it. This would be in the spirit of *securing* individuals at the sufficiency threshold. In this case, however, the emissions threshold is above the sufficiency threshold and the negative thesis does not apply.

What we are left with then is a sphere of healthcare emissions which are permissible. In my view, these GHG emissions are exempt from mitigation burdens because of the role that healthcare plays in securing social justice. But at the very least these GHG emissions should be treated differently in healthcare's overall mitigation strategy. For instance, these emissions should be addressed later with GHG emissions above the emissions threshold tackled first. Outside of this sphere of permissible emissions, however, any remaining healthcare emissions are liable to mitigation, and it is fair for healthcare to shoulder the associated costs and burdens. So, healthcare's GHG emissions are reduced both by applying the test as to whether the emissions are necessary to secure sufficiency but also that emissions are reduced as far as can reasonably expected for healthcare.

Before concluding, I'd like to highlight some commonalities between my approach and a modified ability to pay principle. Ability to pay is a popular way to allocate mitigation burdens and is the main rival to polluter pays which was mentioned in the introduction.^41^ Ability to pay may be thought of as a capacity‐based principle, where mitigation responsibilities are allocated on the basis of agent's capacity to fulfil these responsibilities. In the main, ability to pay equates to the wealthy paying as they are seen as having the greatest capacities and surplus resources to reduce GHG emissions.

When it comes to wealth as the primary marker for being allocated mitigation responsibilities, it is not immediately obvious whether healthcare has the relevant ability to pay. Healthcare systems may have significant sums of money at their disposal, but this tends to be earmarked for providing healthcare. However, if we think of ability to pay in terms of the simple question of ‘what actions can healthcare systems take to reduce their emissions?’ then healthcare does clearly have numerous capacities to minimise emissions. Everything from changing prescribing patterns and moving away from volatile anaesthetic gases to electric ambulance fleets and switching energy supplies shows that there are manifold opportunities for healthcare to reduce emissions without needing to enquire as to whether healthcare is wealthy. Here I have argued that healthcare should be especially inclined to address their emissions where they are not the minimum emissions necessary to secure sufficient health for a decent life. We might think of instances where healthcare emissions are the minimum necessary to secure a threshold of enough as a case when healthcare has an *in*ability to pay, since this would threaten individuals just entitlements and be unfair. We could then think of the emissions threshold as treating some healthcare emissions differently to others according to when healthcare does and does not have an ability to pay.

## 4 CONCLUSION

In this paper, I have sought to integrate two issues. One is to bring together both the benefits offered by healthcare but also the burdens of GHG emissions reduction. Second, is to provide a common basis for determining both the emissions target for healthcare and its fair share of mitigation burdens. Sufficientarianism, I have argued, offers a method of distributing both benefits and mitigation burdens in healthcare that can meet these challenges of integration. I have defended the idea that establishing a sufficientarian threshold of securing enough health can be used to derive a sphere of permissible emissions that are exempt from mitigation burdens. In this way, an ideal of sufficiency offers a theory of distributive justice that can account for both healthcare benefits and mitigation burdens whilst providing a common normative foundation for answering what emissions target healthcare should aim for and their fair share of mitigation burdens.

My goal was to sketch an argument for how sufficiency can answer these important questions of distributive justice. One key issue is how to marry an emissions threshold for healthcare, based on the healthcare's position in fulfilling social justice, with global emissions targets that address issues in global and intergenerational justice. Furthermore, for this account to be suitable in practice, there are complex empirical questions of how to derive an emissions budget from the sufficiency threshold. Sufficiency nevertheless holds promise in guiding the transition to low carbon healthcare.

## CONFLICT OF INTEREST STATEMENT

The author declares no conflicts of interest.

## Footnotes

1 Manfred, L., Malik, A., Li, M., Fry, J., Weisz, H., Pichler, P. P., Chaves, L. S. M., Capon, A., & Pencheon, D. (2020). The environmental footprint of health care: A global assessment. *The Lancet Planetary Health*, 4(7), e271–e279.

2 I note that some may object to my framing of these actions to address climate change as ‘burdens’. I address this concern in the next section and consider whether mitigation is burdensome or whether it is actually better seen as an investment.

3 I thank an anonymous peer reviewer for pressing me on this issue.

4 Romanello, M., Napoli, C. D., Green, C., Kennard, H., Lampard, P., Scamman, D., Walawender, M., Ali, Z., Ameli, N., Ayeb‐Karlsson, S., Beggs, P. J., Belesova, K., Berrang Ford, L., Bowen, K., Cai, W., Callaghan, M., Campbell‐Lendrum, D., Chambers, J., Cross, T. J., … Costello, A. (2023). The 2023 report of the Lancet Countdown on health and climate change: The imperative for a health‐centred response in a world facing irreversible harms. *The Lancet*, *402*(10419), 2346–2394.

5 Naylor, C., & Appleby, J. (2013). Environmentally sustainable health and social care: Scoping review and implications for the English NHS. *Journal of Health Services Research & Policy*, *18*(2), 114–121.

6 Salas, R. N., Maibach, E., Pencheon, D., Watts, N., & Frumkin, H. (2020). A pathway to net zero emissions for healthcare. *Bmj*, *371*, m3785.

7 Parker, J. (2023). Barriers to green inhaler prescribing: Ethical issues in environmentally sustainable clinical practice. *JME*, *49*, 92–98.

8 Shelton, C. L., Knagg, R., Sondekoppam, R. V., & McGain, F. (2022). Towards zero carbon healthcare: Anaesthesia. *BMJ*, *13*, 379; Wilkinson, A., & Woodcock, A. (2022). The environmental impact of inhalers for asthma: A green challenge and a golden opportunity. *British Journal of Clinical Pharmacology*, *88*(7), 3016–3022.

9 Singer, P. (2010). One atmosphere. In S. Gardiner, S. Caney, D. Jamieson, & H. Shue (Eds.), *Climate ethics: Essential readings* (pp. 181–199). OUP; Broome, J. (2012). *Climate matters: Ethics in a warming world*. W. W Norton and Company; Caney, S. (2009). Justice and the distribution of greenhouse gas emissions. *Journal of Global Ethics*, *5*(2), 125–146; Torpman, O. (2019). The case for emissions egalitarianism. *Ethical Theory and Moral Practice*, *22*(3), 749–762; Vanderheiden, S. (2008). *Atmospheric justice: A political theory of climate change*. Oxford University Press.

10 Intergovernmental Panel on Climate Change. (2023) *6th assessment report* (B. 1.3. p. 10). https://www.ipcc.ch/report/ar6/wg3/downloads/report/IPCC_AR6_WGIII_SPM.pdf.

11 Broome, J. (2012). *Climate matters: Ethics in a warming world* (p. 69). W. W Norton and Company; See: Blomfield, M. (2013), Global common resources and the just distribution of emission shares. *Journal of Political Philosophy*, *21*, 283–304. for an opposing view on this characterisation of atmospheric absorptive capacity.

12 Meyer, L., & Roser, D. (2006). Distributive justice and climate change. The allocation of emission rights. *Analyse & Kritik*, *28*(2), 223–249.

13 Cohen, G. A. (1989). On the currency of egalitarian justice. *Ethics*, *99*(4), 906–944.

14 Fankhauser, S., Smith, S. M., Allen, M., Axelsson, K., Hale, T., Hepburn, J. C., Kendall, M., Khosla, R., Lezaun, J., Mitchell‐Larson, E., Obersteiner, M., Rajamani, L., Rickaby, R., Seddon, N., & Wetzer, T. (2022). The meaning of net zero and how to get it right. *Nature Climate Change*, *12*, 15–21.

15 World Health Organization. (2023). *Alliance for transformative action on climate and health*. https://www.who.int/initiatives/alliance-for-transformative-action-on-climate-and-health/country-commitments.

16 Welton, S. (2022) Neutralizing the atmosphere. *Yale Law Journal*, *132*(1), 1–56.

17 Sue‐Chue‐Lam, C., Bhopal, A., Parker, J., & Xie, E. C. (2024). Net Zero is not enough: Ratcheting ambition for sustainable health systems through Reduce and Support. *BMJ Glob Health*, *8*(Suppl 3), e014617. https://doi.org/10.1136/bmjgh-2023-014617.

18 Schulan, A., Tank, L., & Baatz, C. (2023). Distributive justice and the global emissions budget. *WIREs Climate Change*, *14*, e847.

19 Caney, S. (2009). Justice and the distribution of greenhouse gas emissions. *Journal of Global Ethics*, *5*(2), 125–146.

20 Bhopal, A., & Norheim, O. F. (2023). Fair pathways to net‐zero healthcare. *Nature Medicine*, *29*, 1078–1084.

21 Frankfurt, H. (1987) Equality as a moral ideal. *Ethics*, *98*(1) 21–43.

22 Casal, P. (2007). Why sufficiency is not enough. *Ethics*, *117*(2), 296–326.

23 Shields, L. (2020). Sufficientarianism. *Philosophy Compass*, *15*(11), 1–10.

24 Ibid.

25 It could be pointed out that other theories of distributive justice could similarly set an emissions target in the same way as I am describing for sufficientarianism here. The egalitarian, for example, could claim there is some threshold of emissions that is compatible with healthcare systems fulfilling the demands of egalitarian health justice, for instance. A prioritarian might make a similar claim but derive the emissions threshold from prioritarian concerns. Whilst any number of theories of distributive justice could be used to set an emissions threshold, I defend sufficientarianism in the next section as I argue this is better at ensuring that mitigation burdens for healthcare systems are fairly distributed.

26 Timmer, D. (2024). The sufficiency theory of justice and the allocation of health resources. *Bioethics*, *38*, 796–802. https://doi.org/10.1111/bioe.13338; See also, Timmer, D. (2021). Thresholds in Distributive Justice. *Utilitas*, *33*(4), 422–441.

27 Casal, P. (2007). Why sufficiency is not enough. *Ethics*, *117*(2), 296–326; Huseby, R. (2020). Sufficiency and the threshold question. *Journal of Ethics*, *24*, 207–223; Axelsen, D. V., & Nielsen, L. (2015). Sufficiency as freedom from duress. *Journal of Political Philosophy*, *23*, 406–426. https://doi.org/10.1111/jopp.12048; Axelsen, D., & Nielsen, L. (2016). Essentially enough: Elements of a plausible account of sufficientarianism. In F. Carina & A. Rid (Eds.), *What is enough? Sufficiency, justice, and health*; Benbaji, Y. (2005). The doctrine of sufficiency: A defence*. Utilitas*, *17*(3), 310–332.

28 Caney, S. (2009). Justice and the distribution of greenhouse gas emissions. *Journal of Global Ethics*, *5*, 125–146.

29 Vanderheiden, S. (2008). *Atmospheric justice: A political theory of climate change* (p. 243). Oxford University Press.

30 Crisp, R. (2003). Equality, priority, and compassion. *Ethics*, *113*(4), 745–763, p. 762. https://doi.org/10.1086/373954.

31 Axelsen & Nielsen, op. cit. note 27, p. 102.

32 A second, way that philosophers avoid arbitrary thresholds is to propose multiple thresholds. (see: Benbaji, Y. (2005). The doctrine of sufficiency: A defence. *Utilitas*, *17*(3), 310–332; Huseby, R. (2020). Sufficiency and the threshold question. *Journal of Ethics*, *24*, 207–223.) By having more than one threshold, say a higher and a lower threshold, the positive and negative thesis can be more easily justified and appear less arbitrary. One could adopt the positive thesis below the lower threshold and the negative thesis above a higher threshold with further distributive principles used between these thresholds, for instance.

33 Timmer, D. (2024). The sufficiency theory of justice and the allocation of health resources. *Bioethics*, *38*, 796–802. https://doi.org/10.1111/bioe.13338

34 Boorse, C. (1977). Health as a theoretical concept. *Philosophy of Science*, *44*(4), 542–573; Boorse, C. (1975). On the distinction between disease and illness. *Philosophy & Public Affairs*, *5*, 49–68; Nordenfelt, L. (2007). The concepts of health and illness revisited. *Medicine, Health Care and Philosophy*, *10*, 5–10.

35 For an example of this approach see: Ram‐Tiktin, E. (2012). The right to health care as a right to basic human functional capabilities. *Ethical Theory and Moral Practice*, *15*(3), 337–351; See also Venkatapuram, S. (2013). *Health justice: An argument from the capabilities approach*. John Wiley & Sons.

36 Duus‐Otterström, G. (2023). Subsistence emissions and climate justice. *British Journal of Political Science*, *53*(3), 919–933.

37 Shue, H. (1993). Subsistence emissions and luxury emissions. *Law & Policy*, *15*, 39–60. Shue builds on the idea of ‘survival emissions’ widening this to subsistence, see: Agarwal, A., & Narain, S. (1991). *Global warming in an unequal world: A case of environmental colonialism*. Centre for Science and Environment.

38 Duus‐Otterström, G. (2023). Subsistence emissions and climate justice. *British Journal of Political Science*, *53*(3), 919–933.

39 I thank Bridget Pratt for pushing me on this point.

40 I am grateful to Thomas Schramme for drawing my attention to this example.

41 Caney, S. (2005). Cosmopolitan justice, responsibility, and global climate change. *Leiden Journal of International Law*, *18*(4), 747–775; Page, E. A. (2008). Distributing the burdens of climate change. *Environmental Politics*, *17*(4), 556–575.

